# An overview of occult hepatitis B infection (OBI) with emphasis on HBV vaccination

**DOI:** 10.1016/j.heliyon.2024.e37097

**Published:** 2024-08-28

**Authors:** Sara Delghandi, Ramin Raoufinia, Sahar Shahtahmasbi, Zahra Meshkat, Hamed Gouklani, Aida Gholoobi

**Affiliations:** a Medical Genetics Research Center, Mashhad University of Medical Sciences, Mashhad, Iran; b Division of Immunology and Genomic Medicine, Center for Cancer Immunotherapy and Immunobiology, Graduate School of Medicine, Kyoto University, Kyoto, Japan; c Noncommunicable Diseases Research Center, Neyshabur University of Medical Sciences, Neyshabur, Iran; d Metabolic Syndrome Research Center, Mashhad University of Medical Sciences, Mashhad, Iran; e Antimicrobial Resistance Research Center, Mashhad University of Medical Sciences, Mashhad, Iran; f Infectious and Tropical Diseases Research Center, Hormozgan Health Institute, Hormozgan University of Medical Sciences, Bandar Abbas, Iran

**Keywords:** General population, Mother-to-child-transmission, Hepatitis B vaccine, Hepatitis B virus, Chronic hepatitis B

## Abstract

**Background:**

The prevalence of chronic hepatitis B virus (HBV) poses a significant threat to the lives of 257 million individuals globally, potentially resulting in severe outcomes such as liver cirrhosis or hepatocellular carcinoma. Among the existing preventive measures, yeast-derived vaccines have proven to be the most efficacious approach in combatting hepatitis B. Nonetheless, as scientific inquiries focus more on occult HBV infection (OBI) in vaccinated persons and the lingering risk of vertical transmission affecting 10–30 % of babies born to HBsAg-positive mothers, there is a growing apprehension regarding the inability of HBV vaccines to ensure complete immunity. This study aims to offer a more comprehensive understanding of the implications of widespread HBV vaccination initiatives on OBI while tackling the primary limitations associated with current vaccine formulations.

**Methods:**

The exploration was conducted on PubMed, Scopus, and Web of Science databases to pinpoint research on OBI within vaccinated cohorts. A sum of 76 suitable studies was recognized.

**Discussion:**

Multiple studies have documented the occurrence of OBI in fully vaccinated individuals, including both the general population and high-risk groups, such as newborns born to HBsAg-positive mothers. Factors contributing to vaccine failures include low-level anti-HBs antibodies, high maternal viral loads in mother-to-child transmission cases, as well as the presence of vaccine escape mutants and heterologous HBV genotypes. However, further research is needed to precisely understand the impact of active immunization on the emergence of OBI in vaccinated populations. Nonetheless, it is apparent that the advancement of more effective HBV vaccines could potentially lead to the extinction of HBV.

## 1 Introduction

Occult hepatitis B infection (OBI) refers to the presence of replication-competent hepatitis B virus (HBV) DNA in the liver, even when HBV DNA is not detectable in the blood using standard assays. During this phase of chronic HBV infection, the HBV genomes exist as episomal covalently closed circular DNA (cccDNA) and exhibit low replication activity. Consequently, HBV DNA in serum or plasma is intermittently detectable, often at levels below 200 International Units (IU)/mL. The prevalence of detectable HBV DNA in the serum of HBsAg-negative carriers varies based on factors such as the population studied, assay sensitivity, and timing of blood sample collection [[1], [2], [3], [4]].

OBI can be categorized as seropositive or seronegative. Seropositive OBI refers to individuals with detectable antibodies against the HBV core antigen (anti-HBc) and/or antibodies against HBsAg (anti-HBs) in their serum, accounting for approximately 80 % of OBI cases. In contrast, seronegative OBI individuals lack all HBV serum markers (including anti-HBc and anti-HBs) but still harbor intrahepatic HBV DNA (and occasionally circulating HBV DNA). The duration of HBsAg positivity before its disappearance can vary significantly in seropositive OBI cases [5].

Primary seronegative occult infection has been observed in woodchuck models following inoculation with a low number of hepadnavirus virions (less than 100). Additionally, some OBI cases may result from HBV genetic variants with S gene mutations, leading to undetectable modified HBsAg despite high serum HBV DNA levels. Although HBV DNA can integrate into the host genome in OBI individuals, these integrated viral sequences do not contribute to HBV replication because the circular HBV genome is disrupted by the integration. The presence of replication-competent HBV DNA remains the key factor in diagnosing OBI [5,6].

### 1.1 Biology of OBI

OBI is characterized by the stability of HBV cccDNA chromatinized episomes within hepatocyte nuclei, allowing long-lasting persistence. Despite the presence of cccDNA, OBI patients do not exhibit detectable HBsAg due to the suppression of HBV gene expression and replication by epigenetic mechanisms and immune control. Studies indicate that OBI patients may have more mutations in the pre-S/S region, potentially affecting HBsAg detection or production. However, cccDNA in OBI remains fully replication competent. Transmission via blood transfusions and organ transplants can lead to overt HBV infection in recipients, and OBI may reactivate during immunosuppression. Host factors likely play a crucial role in OBI development. Low cccDNA concentrations in hepatocyte nuclei result in minimal HBV transcripts and protein expression, leading to HBsAg undetectability [5,7,8]. Epigenetic regulation, including methylation patterns and post-translational histone modifications, may contribute to this phenomenon. Although various epigenetic mechanisms control viral replication, direct evidence in OBI patients remains limited. Additionally, the immune response to HBV indirectly influences OBI, as evidenced by reactivation during immunosuppression [5,[9], [10], [11], [12], [13], [14], [15], [16]].

### 1.2 Diagnosis of OBI

The diagnosis of OBI relies on detecting HBV DNA in blood or liver samples from individuals who test negative for HBsAg. While liver biopsy is the gold standard for detection, it is not commonly used due to its invasiveness. Anti-HBc antibodies are often used in place of HBV DNA detection, but these antibodies may be negative in seronegative OBI cases. Therefore, a combination of HBV DNA and HBsAg detection is necessary for an accurate diagnosis. Inaccurate results may occur due to insufficiently sensitive HBsAg detection assays, leading to misdiagnosis of OBI [17,18].

In recent years, high-sensitivity HBsAg assays such as Lumipulse HBsAg-HQ were introduced with a sensitivity of 0.005 IU/mL, approximately ten times higher than conventional assays (0.05 IU/mL) [19]. These assays can identify HBsAg/anti-HBs complexes more efficiently, enabling them to detect between 1 % and 48 % of samples that are tested negative by previous methods [[20], [21], [22]]. A more recent development is the fully automated Lumipulse Presto HBsAg-HQ with the same sensitivity as HBsAg-HQ. This assay was reported to detect HBsAg in patients with HBV reactivation earlier than conventional assays, showing its potential for early detection [23]. Recent studies have developed ultra-sensitive HBsAg assays with a higher sensitivity of 0.0005 IU/mL to detect low levels of the virus, helping to prevent false negative results. Shinkai et al. found that ultra-sensitive HBsAg assays such as ICT-CLEIA are more effective in detecting HBV reactivation in patients with hematological malignancy undergoing chemotherapy compared to conventional assays [24]. They also observed that ICT-CLEIA detected HBsAg in all patients with reactivation. Two of the 12 patients tested HBsAg-positive even before HBV DNA was detectable.

High sensitivity and ultra-sensitive HBsAg assays have also been instrumental in identifying escape variants in the HBV S gene, which are important for diagnosing OBI accurately. The use of multivalent anti-HBs antibodies in HBsAg assays is recommended to ensure proper detection of these variants [19]. While the evidence available may be limited, it is crucial to acknowledge the potential for false positives in HBsAg results obtained through ultrasensitive methods. Consequently, it is advisable to consider corroborating these findings using alternative assays or HBV DNA testing.

OBI is frequently detected by examining serum samples due to the limited availability of liver biopsy procedures. When OBI is present, the amount of HBV DNA in the serum is typically very low, usually below 200 IU/mL. HBsAg negative individuals with persistent HBV DNA levels under 200 IU/mL are identified as true OBI [25]. Commercial HBV DNA assays typically detect down to 10–20 IU/ml, and consistency across HBV genotypes and subtypes is crucial. Due to low and inconsistent HBV DNA levels in OBI, Testing blood samples from multiple time points and extracting DNA from at least 1 ml of serum or plasma is recommended for OBI diagnosis. Nucleic acid testing (NAT) assays used in blood transfusions have 99.9 % specificity and detect 2–4 IU/ml HBV DNA. However, when multiple donations are pooled, sensitivity reduces due to dilution [26,27].

### 1.3 Serology of OBI

Serological markers are used to classify OBI into seropositive or seronegative cases. Seropositive OBI, which makes up 80 % of cases, is characterized by the presence of anti-HBc and/or anti-HBs in the serum. In contrast, seronegative OBI has neither of these antibodies present, making diagnosis more difficult as serum HBV DNA is the only detectable marker. It is worth noting that primary seronegative occult infection has been observed in woodchucks infected with a low number of virus particles. Detection of positive serum HBV DNA is most likely in individuals who are anti-HBc positive but anti-HBs negative [28,29]. Anti-HBc negative/anti-HBs positive results should be interpreted with caution because they may indicate active/passive immunization unless HBD DNA becomes positive.

### 1.4 Epidemiology of OBI

The global epidemiology of OBI varies due to factors such as assay sensitivity, HBV risk factors, vaccination programs, and liver disease prevalence. Studies on OBI prevalence are often conducted on blood donors and liver disease patients, which may not fully represent the general population. While OBI is more common in regions where hepatitis B is endemic, some Asian and African areas with high HBV prevalence have reported low OBI rates. OBI is more prevalent in high-risk populations, such as injection drug users, individuals with HCV or human immunodeficiency virus (HIV) co-infections, and patients undergoing dialysis [5,[30], [31], [32], [33]]. Higher OBI rates are also seen in patients with liver diseases like hepatocellular carcinoma, and cryptogenic cirrhosis, or those who have undergone liver transplants. Studies have also found OBI in patients with non-alcoholic fatty liver disease, with varying prevalence rates reported. OBI is rarely detected in blood donors, with HBV DNA found in a small percentage of HBsAg-negative/anti-HBc-positive individuals [5,[34], [35], [36]].

### 1.5 HBV transmission by OBI

1) Blood Transfusion

Research has shown that OBI-positive donors can transmit HBV through blood transfusions, leading to the development of hepatitis B in the recipients [[37], [38], [39]]. The risk of transmission from an OBI donor is influenced by several factors, including the amount of transfused plasma, the immune status of the recipient, and the HBV serological status of both parties [37]. More recent studies have indicated a need for more sensitive screening, as the minimum infectious dose of HBV (3.0 IU/mL) is lower than previously estimated (20 IU/mL). To prevent HBV transmission through transfusion, the nucleic acid test (NAT) sensitivity needs to be lowered to 0.15 IU/mL from 3.4 IU/mL [3].

2) Liver Transplantation

There is a recognized risk of HBV transmission from a seropositive OBI liver donor to an HBV-susceptible, seronegative recipient, which can lead to hepatitis B [40,41]. To prevent this, long-term antiviral therapy with nucleos(t)ide (NUC) analogs such as entecavir or tenofovir is recommended. Even so, the occurrence of OBI in the recipient may not be entirely prevented by NUC prophylaxis [42]. Patients who have had a liver transplant due to HBV are at risk of developing OBI in the liver graft, even with antiviral prophylaxis. Therefore, lifelong NUC therapy is recommended for all liver transplant recipients to help prevent the development of OBI [43,44].

3) Mother-to-child transmission

Despite proper active/passive immunoprophylaxis at birth, HBsAg-positive mothers may still transmit OBI to newborns. The detection of anti-HBc, not HBsAg, after the age of one, indicates that although the HBV infection wasn't fully prevented, progression of chronic HBV infection was successfully avoided [3,75,80,90,91,110].

### 1.6 Risk factors for OBI

Identifying risk factors associated with OBI is essential for preventing transmission. Key risk factors include patients with a history of HBV infections, individuals co-infected with HCV or HIV, recipients of organ transplants, blood donors, thalassemia or hemophilia patients, those with cryptogenic hepatitis, cirrhosis, and hepatocellular carcinoma, individuals undergoing hemodialysis, patients treated with medications like lamivudine or interferon, children in regions with high HBV prevalence, and immunocompromised patients receiving biological treatments or chemotherapy (particularly anti-CD20 therapy) [17,45]. The occurrence of OBI is relatively common in patients with HCV co-infection due to shared transmission routes. Various studies reveal that the prevalence of OBI among hepatitis C virus (HCV) patients ranges from 0 to 52 % [46]. OBI prevalence in HCV co-infected patients can be due to the HBsAg gene mutations or low HBV replication. The co-existence of HBV and HCV genomes in the same liver cell could inhibit HBV replication through interference of HCV molecules [47,48]. The presence of OBI in these patients could be associated with more severe liver damage, cirrhosis, and a higher rate of liver cancer [[49], [50], [51], [52], [53]]. However, the clinical impact of OBI on chronic HCV patients remains uncertain [17].

Patients with HIV co-infection are another group susceptible to OBI due to shared transmission routes with HBV. The prevalence of OBI in this group is also variable, ranging from 0 % [54] to 15 % [55]. The impact of this co-infection on clinical outcomes is not well understood [17].

As for blood donors, the prevalence of OBI is very low [56]. However, to prevent HBV transmission through blood transfusion, nucleic acid tests (NAT) have been recommended for their higher sensitivity in detecting HBV DNA [28]. Patients on hemodialysis are at a higher risk of HBV infections. Therefore, routine screening for HBV and OBI is recommended in these patients [17]. Finally, OBI has been identified in patients with cryptogenic liver disease, characterized by an unknown etiology [57]. It is recommended that HBV DNA determination by high-sensitive molecular assays is conducted before the patient develops signs of cirrhosis or liver cancer [28].

**Fig. 1:**
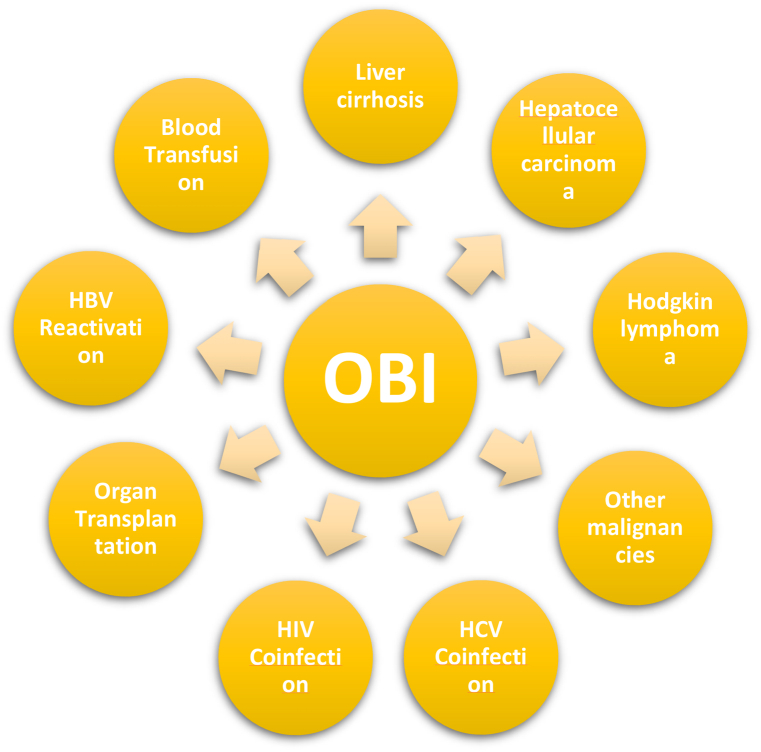
Schematic representation of clinical significance of OBI. HCV: Hepatitis C virus; HIV: Human immunodeficiency virus.

The conditions illustrated in Fig. 1 highlight the important role of HBV vaccination in the prevention of OBI. Along with the national vaccination program, high-risk groups such as healthcare workers and newborns of HBsAg positive mothers have received HBV vaccination since 1993. However, there have been several longitudinal studies involving OBI that have reported the emergence of OBI in vaccinated individuals. The debate has gained fresh prominence, with many arguing that the deficits of current vaccines have led to these vaccine failures and new improved generation of vaccines should be made available worldwide. The purpose of this paper is to provide a short review of recent research into the impacts of the general HBV vaccination program on OBI and to address the major debates about the drawbacks of current vaccines.

### 1.7 Search strategy

The published studies on OBI among vaccinated populations were collected by searching PubMed, Scopus, and Web of Science databases. The search terms included “Hepatitis B″ OR “Hepatitis B Virus” OR “HBV” OR “Chronic Hepatitis B” OR “Hepatitis B Infection” AND “Occult Hepatitis B Virus Infection” OR “OBI” OR “Occult HBV Infection” AND “Hepatitis B Vaccine” OR “Vaccination” OR “Vaccines”. A total of 156 related articles was identified. Finally, 76 articles were found to be eligible for this literature review. Eligibility criteria include (I) studies reporting OBI in vaccinated populations; (II) nested PCR used to detect HBV DNA; (III) sufficient information for the analysis; (IV) only patients who have negative-HBsAg; (V) no other concomitant liver diseases.

### 1.8 HBV vaccination: from development to shortcomings

Active immunization is an important landmark in the prevention of hepatitis B diseases. Two generations of vaccines have been developed since 1969 when Blumberg suggested that a hepatitis vaccine can be generated by the purification of AuAg from human serum [9]. The first approach involved extracting HBsAg from chronic HBV carriers' plasma. The plasma-derived vaccines, nonetheless, were unsatisfactory for a variety of reasons. An important cause of concern was that, over the years, the administration of vaccines would reduce the number of HBsAg carriers [[58], [59], [60]]. In other words, the potential resource of vaccines would gradually become unavailable. Thus, when yeast-derived vaccines were developed in the 1980s, many countries ceased to use first-generation vaccines and replaced them with those of the second generation [61]. By the end of 2018, 189 countries had introduced national hepatitis B vaccination for infants, and HBV vaccination coverage was reported to be 84 % after the third dose. The implementation of a universal HBV immunization program has proved to be effective in the reduction of both acute HBV infections and carrier rates among vaccinated cohorts [[61], [62], [63]]. Long-term follow-up studies in Taiwan, a previously hyperendemic area, demonstrated a considerable decline from 9.8 % in 1984 to 0.7 % in 1999 in the prevalence of HBsAg among children younger than 15 years of age [64]. Likewise, following vaccination, childhood prevalence of HBsAg in Gambia, Malaysia, and Hawaii showed a clear decline to less than 1 % by 2004 [[65], [66], [67]].

HBV vaccine is the first example of cancer prevention by vaccination in humans [68]. In Taiwan, a study on the incidence of hepatocellular carcinoma in children 6–9 years of age showed a significant decline from 0.52/10^5^ to 0.13/10^5^ after the implementation of the universal vaccination program. Although the administration of recombinant vaccines has dramatically reduced the incidence of new HBV infections and subsequent development of hepatocellular carcinoma [[69], [70], [71]], there is now much concern about their shortcomings. In fact, vaccine responsiveness varies from person to person depending on gender and age [72]. Some factors contributing to impaired response include obesity and smoking as well as various conditions, such as diabetes, hemodialysis, kidney disease, celiac disease, and HIV infection, which cause a weaker response due to the impaired immune system [72]. Four weeks after the last dose of vaccine, recipients with anti-HBs levels less than 10 IU/mL are considered to be "unprotected non-responders" and those with titers higher than 10 IU/L are believed to be fairly protected against HBV disease [73,74]. Since the first vaccines were developed, it has been known to scientists that vaccinated individuals are not completely immune against HBV infection. Nevertheless, the recent findings regarding OBI have led to more uncertainty about the effectiveness of current vaccines.

### 1.9 The emergence of OBI in vaccinated cohorts

In recent years, several asymptomatic breakthroughs have been reported in fully vaccinated people. Therefore, occult HBV could no longer be neglected in vaccinated populations. In Taiwan, monitoring for 25 years has shown a 3.1 % increase in the frequency of OBI (HBsAg-Negative, anti-HBc-positive subjects) following universal infant immunization [75]. In a recent Chinese study carried out on 2028 vaccinated blood donors, 20 OBI cases were reported. The authors pointed out that vaccination failed to prevent HBV infection in a considerable proportion of donors [76]. In a recent study, Egyptian researchers found evidence of HBV breakthrough infection in 0.39 % of fully vaccinated children. In the same literature, authors reported anti-HBs >100 IU/L in five children, mentioning that these successfully vaccinated individuals were later exposed to HBV [77]. Table 1 enumerates various studies that document Occult HBV Infection in individuals who have been vaccinated.

**Table 1:** Lists several studies reporting OBI in vaccinated populations.

Authors	Year	Number of study samples	OBI (%)	Anti-HBc (%)	Country
Chen et al. [78]	2002	126	ND^a^	0.9	Taiwan
Liu et al. [79]	2005	467	2.35	0	Taiwan
Mu et al. [80]	2008	46	10.86	ND^a^	Taiwan
Xu et al. [114]	2010	2919	4.2	100	China
Chakvetadze et al. [82]	2011	100	2	6	Africa
Chiaracul et al. [83]	2011	56	7.14	64.2	Thailand
Pande et al. [84]	2013	213	42	ND^a^	India
Su et al. [85]	2013	183	4.92	33.87	China
Elrashidy et al. [86]	2014	170	0	0	Egypt
Gessoni et al. [87]	2014	21	1(transient)	100	Italy
Liu et al. [88]	2014	210	0	1.42	China
Hsu et al. [75]	2015	334	4.8	100	Taiwan
Sadeghi et al. [112]	2015	17	5.88	11.76	Iran
Foaud et al. [90]	2015	64	1.56	1.56	Egypt
Amponsah-dacosta et al. [91]	2015	53	66	40	South Africa
Borzooy et al. [89]	2015	120	3.3	0	Iran
Aghakhani et al. [92]	2015	92	2.17	0	Iran
Kim et al. [93]	2015	87	7.69	ND	Korea
Wang Z et al. [94]	2016	475,538	0.02	ND	China
Lai et al. [95]	2016	705	5.39	0	Taiwan
Lu et al. [96]	2016	158	20.3	6.3	China
Morsica et al. [97]	2017	16	37.5	100	Italy
Yokoyama et al. [98]	2017	158	1.3	ND	Japan
Zhou et al. [99]	2017	77	36.4	ND	China
Rodríguez Lay et al. [100]	2017	32	3.12	15.6	Cuba
Tang X et al. [101]	2018	14937	0.12	20.9	China
Zhuge et al. [102]	2020	327	3.1	14.1	China
de Souza Marques et al. [103]	2022	1083	0.5	32.8	Brazil

a ND: Not Determined.

### 1.10 The burden of mother-to-child-transmission (MTCT)

A primary concern of WHO is the prevention of HBV infection in children before the age of five. Infants born to HBsAg-positive mothers who acquire the infection via vertical transmission play an important role in maintaining the endemicity of HBV infection. Up to 90 % of the Infected newborns tend to develop chronic HBV infection, a leading cause of cirrhosis and HCC, with a 15–25 % risk of death [104]. In order to prevent mother-to-child transmission, a first dose of vaccination combined with hepatitis B immune globulin (HBIG) is recommended within 24 h of birth, followed by 2–3 further doses, one month and six months later [72]. However, recent evidence suggests that even a combination of passive and active immunization could not guarantee complete protection against perinatal transmission, as 10–30 % of newborns develop chronic HBV infection. What is not yet well understood is why these failures happen [105]. A possible explanation for this might be the extremely high viral load in the blood of HBeAg-positive mothers. Several studies have revealed that occult HBV infection happens rather frequently among these young vaccinees. In China, Su et al. investigated the prevalence of OBI in vaccinated children born to HBsAg-positive mothers. Approximately 4.9 % of the infants were reported to have OBI. The authors identified three factors as being potentially important in the emergence of occult HBV infection: First, OBI-positive infants had a significantly higher percentage of maternal viral loads (almost 67 %) in comparison to OBI-negative ones. Second, six out of nine children with OBI had low-level anti-HBs, which contribute to limited neutralizing capacity, and three of them were anti-HBs-negative. Subsequently, vaccine escape mutants (S143L mutation) were reported in four OBI cases [85]. In many African countries with a high prevalence of HBV infection, children are vaccinated at six weeks of age rather than at birth. HBV prevalence among children in Africa is more than two and a half times greater than among those in other regions. As of 2021, only 17 % of newborns in Africa received the timely hepatitis B birth dose vaccine. This delay in vaccination could potentially contribute to the prevalence of OBI in the region [[106], [107], [108]]. Some researchers hold the view that the use of HBIG in vaccination programs can trigger not only immune pressure but also the selection of vaccine variants [77,84,109]. According to the findings of a 2013 study, occult HBV was more frequent in infants who received HBIG than those who did not [84]. The results of several studies on the prevalence of OBI in MTCT cases are summarized in Table 2.

**Table 2:** OBI cases in children born to HBsAg positive mothers.

Authors	Year	Number of study samples	OBI positive cases (%)	Vaccine^a^	HBIG^b^
Mu et al. [80]	2008	46	10.86	yes	ND^c^
Chakvetadze et al. [82]	2011	100	2	yes	yes
Shahmoradi et al. [110]	2012	75	28 (21.33 transient)	yes	yes
Pande et al. [84]	2012	128	group 1: 35.15	yes	yes
		131	group 2: 33.58	yes	no
Su et al. [85]	2013	186	4.91	yes	ND
Liu et al. [88]	2014	210	0	yes	yes
Foaud et al. [90]	2015	64	1.56	yes	yes
Lu et al. [96]	2016	158	20.25 (15.82 transient)	yes	yes
Zhou et al. [99]	2017	77	36.36 (10.38 transient)	yes	yes
Ghaziasadi et al. [111]	2020	660	16	yes	yes

a The status of receiving HBV vaccine at birth.

b The status of receiving HBIG at birth.

c ND: Not Determined.

Interestingly, follow-up studies showed that in some vaccinated infants, HBV DNA detected in the early stage of life was not persistent, which means it disappeared a few months after the initial detection. In 2012, an Iranian cross-sectional report estimated the prevalence of OBI in immunized children born to HBsAg-positive mothers to be 28 % [110]. Three years later, Sadeghi et al. studied the persistence of HBV DNA in 17 children who were identified to be OBI-positive in the previous study. Surprisingly, they found that 94 % of children had become negative for HBV DNA after 54 months [112]. Similarly, other authors (Lu et al., 2016; Zhou et al., 2017) have reported transient OBI, which is characterized by the short-term persistence of HBV DNA, in a number of vaccinated children [96,99]. Pande et al. have speculated the successful clearance of occult HBV infection in some infants is due to their adequate anti-HBs titers. In their study, 36 % of infants with high anti-HBs levels lost OBI after 18 weeks, whereas only 5 % of those with low-level anti-HBs cleared the infection [84]. Together, these studies highlight the need for follow-up detection of HBV DNA in MTCT cases already known to be OBI-positive. To tackle the issue of MTCT, it is recommended to use Tenofovir instead of Lamivudine or Telbivudine for antiviral therapy in mothers. Tenofovir is safer for the fetus and less likely to cause drug resistance compared to the other options [31,62,81].

So far, we discussed the emergence of OBI in general vaccinated populations as well as infants born to HBsAg-positive mothers, a population at high risk. One question that needs to be asked, however, is whether these failures have something to do with the inefficiency of current HBV vaccines.

### 1.11 Possible sources of vaccine failures

1) HBV genotypes

If we now turn to the shortcomings of vaccines, HBV genotypes could be an important source of the problem. The serological subtype represented in current far-reaching recombinant vaccines is HBV genotype A2 [71,113]. From Table 3, we can note that most OBI cases have been reported in high-prevalence countries such as China, Thailand, and Taiwan. However, In China, for instance, the most prevalent HBV genotypes are B and C. Ten known HBV genotypes distribute inconsistently in different continents [113]. Although A2-based vaccines are assumed to provide cross-protection against other HBV subgenotypes [114], a recent study on blood donors of the American Red Cross has challenged the validity of this claim. In 2008, nucleic acid testing, which is a sensitive molecular method for detecting small amounts of DNA or RNA in blood donations, was performed on 2.14 million donors. Among seronegative donations, nine donors (three unvaccinated and six vaccinated individuals) were identified as HBV DNA positive. Five of six vaccinated donors with Anti-HBs levels of 10–100 IU/L were reported to have non-A genotypes. The only case with genotype A was discovered to have Anti-HBs<10 IU/L and, therefore, considered to be nonimmune. On the other hand, the dominant genotype in all three unvaccinated donors was A2 [115]. In the mentioned study, the main reason for vaccine failure is likely to be the heterologous HBV genotypes carried by the majority of infected donors, and this calls into question some past assumptions about the effectiveness of current vaccines for non-A genotypes.

2) Vaccine escape mutants

**Table 3:** Articles represented OBI cases in children born to HBs Ag-positive mothers who had received the Hepatitis B (HB) vaccine at birth.

Authors	Year	OBI cases (%)	Number of mutations in OBI cases(n)	Mutation type
Mu et al. [80]	2008	10.86	ND	no G145R but pre-S deletion (C139S vaccine escape mutant was found).
Xu et al. [114]	2010	14.2	4	G145R
Shahmoradi et al. [110]	2012	28	10	G145R
Su et al. [85]	2013	94.92	4	S143l
Hsu et al. [75]	2014	2.67	5	Pre-S1 variants with wild-type S region
Kim et al. [93]	2015	7.69	6	XDel8
Foaud et al. [90]	2015	1.56	ND*	There are no reported HBV S gene mutations in these patients
Amponsah-dacosta et al. [91]	2015	366	2	HBV S gene variant including diagnostic mutation
Aghakhani et al. [92]	2015	22.17	1	145 (G145R)
Sadeghi et al. [112]	2015	5.88	1	G145R
Yokoyama et al. [98]	2017	21.3	1	G145R

ND: Not determined.

Vaccine escape mutants are probably another drawback of HBV vaccines that have been overlooked. The most important antigenic region of HBsAg is “a” determinant that serves as the target of antibodies elicited by HBV vaccines. Mutations altering the primary structure of "a" determinant and antigenicity of HBsAg may hinder vaccine-induced antibodies from neutralizing the virus [104]. The most well-known immune escape mutant in the "a" determinant is a replacement of glycine by arginine or G145R, which was first discovered in an Italian child born to an HBsAg carrier mother despite having received both active and passive immunization [104]. As the mutants could lack specific epitopes to which vaccine-induced antibodies are directed, they are selected from the viral population, and the fact that antibodies fail to neutralize them leads to vaccine inefficiency [116]. To determine the prevalence of HBsAg escape mutants, Hsu et al. compared the results of six surveys conducted between 1984 and 2009. They reported an approximately 15 % rise in the prevalence of escape mutants 15 years after the universal vaccination program in Taiwan, which was higher in fully vaccinated individuals compared with unvaccinated ones [117].

Interestingly, vaccine-escape mutants, in many cases, have been reported to be associated with the emergence of OBI in people who received complete vaccinations (Table 3). In fact, one of the underlying mechanisms of OBI is the occurrence of mutations in various HBV genome regions leading to the non-detection of HBsAg and HBsAg negativity [116]. Almost every paper that has been written about the prevalence of OBI in the vaccinated population includes a section relating to mutation analysis. An example of this is the study carried out by Shahmoradi et al. in which 62 % (13/21) of OBI-positive vaccinated children were reported to have at least one mutation, and 10 OBI isolates were reported to contain G145R mutations [110]. As can be seen from Table 3, only a small number of OBI-positive individuals carry mutations, which shows that escape mutants are unlikely to be the main cause of occult infection breakthroughs. However, escape mutants are not insignificant. In 2013, Feeney et al. reported a case of reactivated OBI in a patient who had undergone cytotoxic chemotherapy for follicular lymphoma. The patient had a history of a long-term stay in Papua New Guinea, probably where he acquired HBV despite having received HB vaccination and remaining seronegative for HBsAg and Anti-HBc. In further analysis, the HBV genotype was identified as D4, and several mutations were detected including three in the "a" determinant region (V128A, G130R, S143L) [118]. This case highlights that occult infection caused by escape mutants can be reactivated following immunosuppression. Besides, it is a good example of how, in some cases, hepatitis B vaccines fail to protect against both non-A genotypes and escape mutants. The 2008 study on American Red Cross blood donations, discussed in the previous section, also reported that one of the OBI-positive donors infected with a non-A2 strain was carrying the G145R vaccine-related escape mutation [115]. As mentioned above, some escape mutants are vaccine induced. However, a large number of mutations leading to OBI may occur naturally [104,116], which questions the need for better vaccines, as the current ones are unable to provide complete protection against an abundance of mutated variants.

## 2 Discussion

Active immunization is a key strategy for the prevention of HBV infection. To quote a 2017 WHO report: "In 2015, the global coverage with the third dose of hepatitis B vaccine reached 84 %". This early success led to an outstanding reduction in the incidence of chronic HBV infection in the first five years of life. Thus, many experts brought up the prospect that viral hepatitis could be eliminated by 2030, and this would be possible by a 90 % and a 65 % reduction in new infections and mortality, respectively [119]. However, acknowledging the emergence of occult HBV infection in vaccinated populations, will we accomplish this goal of WHO ten years from now?

OBI is generating considerable interest because it is established to have a role in several clinical conditions. An increasing number of studies have found that OBI might be transmitted by blood transfusion and organ transplantation. It could also be reactivated in patients undergoing immunosuppression [120].

As explained earlier in this paper, we have good evidence that occult HBV infection can occur in fully vaccinated individuals. In fact, some experts have questioned whether current vaccines can eventually favor the emergence and increase of OBI [75,121]. However, further work is required to establish this. What we know is vaccines fail to provide an adequate level of immunity in a minority of people, and this is not negligible, at least for individuals at high risk. Pande et al. showed that in MTCT cases, infants with adequate anti-HBs titers successfully cleared the occult infection after a few months, while infants with a weak immune response to vaccines did not [76]. This example emphasizes the need for more immunogenic vaccines. On the contrary, in 2006, a vaccinated thrombopheresis donor with anti-HBs levels of more than 1000 IU/L was reported to have OBI. The infecting virus was identified as genotype D, a genotype different from that vaccine, and it contained a P120T mutation [122]. This case highlights two deficits of current vaccines we discussed earlier in this paper: HBV genotypes and escape mutants.

Studies on blood donors in China indicate a need for a booster dose to fight occult HBV infections among young, vaccinated blood donors. Isolated anti-HBc, anti-HBc, and anti-HBs, and even anti-HBs alone accompanied by sporadic HBV DNA detection suggest potential vaccine failure, possibly due to waning immunity with low anti-HBs levels 10–20 years post neonatal vaccination or non-adherence to the vaccine regimen. There is a proposal for booster vaccination during adolescence, though this is currently debated and not widely recommended [94,101].

Overall, there would seem to be a definite need for improvement of the current vaccines. One strategy is the production of vaccines that cover the major HBV genotypes. Additionally, some experts have considered the third generation of vaccines produced with preS epitopes combined with immunogenic HBV core particles as a promising option [71]. Two third-generation vaccines, Heplisav-B and PreHevbrio, were developed to enhance seroprotection and minimize vaccine doses [123]. Heplisav-B, also known as Hep-CpG, combines the small S protein with a unique cytosine phosphoguanine (CpG) adjuvant and was approved in the U.S. in 2017. Heplisav-B's two-dose regimen is faster than standard vaccines and long-lasting, particularly in low-response groups [[124], [125], [126]]. PreHevbrio, originally known as Sci-B-Vac, was approved in 2021 and includes the small S protein, plus large and middle proteins. PreHevbrio's three-dose regimen demonstrated similar seroprotection rates to standard vaccines in adults [127,128]. Future studies could explore how the application of third-generation vaccination affects the prevalence of OBI.

Although OBI is a complex entity that is proven to have several clinical implications (Fig. 1), it has been frequently overlooked in discussions of HBV vaccination. In fact, only a limited number of studies have aimed at investigating HBV DNA in HBsAg-negative subjects to determine the prevalence of OBI after universal vaccination programs. Moreover, the available literature on the prevalence of OBI in the vaccinated population suffers from several drawbacks. First of all, most of the studies have been carried out in high-endemic countries such as Taiwan, China, etc. (Table 1). As a result, our knowledge about regions with low to moderate endemicity is limited, and further research in these countries is advisable. In addition, vaccine coverage and the distribution of HBV genotypes in different countries should be taken into account. Another issue is the contrasting results from previous studies (Table 1), which may be due to the differences in methodology and sensitivity of the tests [112].

Recent studies on MTCT cases offer contradictory data about the occurrence of OBI in HB-vaccinated newborns (Table 2). However, a few researchers reported that a considerable number of infants initially characterized as OBI-positive cleared HBV DNA a few months after being diagnosed, which means they had transient OBI [84,99,100,112]. This type of OBI is rare and difficult to detect [25]. Unfortunately, most of the previous studies on the prevalence of OBI in vaccinated infants lacked follow-ups to see if the occult infection was persisting in them. Hence, further cohort studies that investigate serial follow-ups in certain intervals will need to be undertaken. In addition, to determine whether OBI in infants has originated from their HBsAg-positive mothers, it is crucial to perform sequencing and phylogenetic analysis. A study in southern China showed that among 28 children classified as OBI, only 6 had HBV genotypes closely related to those of their HBsAg carrier mothers [99]. As other sources of infection rather than the mothers are also possible [89], a key problem with many of the previous studies on these cases is that they did not consider a viral and phylogenetic analysis of HBV strains infecting mother-child pairs to compare them.

The management of MTCT cases remains a major challenge since there is a lack of consensus regarding whether these infants should receive a combination of the HB vaccine and HBIG or the vaccine alone. According to a recent meta-analysis, neonates of HBsAg-positive mothers who receive the HB vaccine alone or as effectively protected against overt infection as those who receive HBIG as well [129]. On the other hand, some reports have demonstrated that HBIG can favor occult HBV infection since it can contribute to immune pressure, which results in the selection of mutations in the virus [75,84]. A further study with more focus on the impact of HBIG in MTCT subjects is therefore suggested.

Another aspect of research on vaccinated children that is extensively disregarded is the possibility of transmission from occult infected mothers. Pregnant women with occult HBV infection are often missed in the routine screening methods, and they tend to be classified as healthy individuals. Therefore, a less effective HBV vaccination protocol would be administered for their newborns [130,131]. Although there is a great deal of evidence for the clinical significance of OBI, most of these studies did not determine whether OBI-positive people were vaccinated or not. Furthermore, we have evidence indicating OBI can lead to clinically significant acute hepatitis B in unvaccinated people [99,115,132]; However, the maintenance of occult infection and the development of clinically important disease in OBI-positive vaccinated individuals remains elusive, due to the lack of serial follow-ups in the previous studies.

Subsequently, few studies have been published on the prevalence of OBI in other high-risk groups of vaccinated populations. Approximately three million healthcare workers each year are at the exposure to infectious blood and body fluids studies, mostly as a consequence of needle-stick injuries [133]. Thus, the prevalence of occult infected health care workers after vaccination is particularly an important issue to resolve in the future.

In conclusion, despite the major success of the universal HBV vaccination program, OBI is a prospective obstacle to achieving the ultimate goal of WHO, which is the eradication of viral hepatitis by 2030. More retrospective cohort research needs to be undertaken in countries with low to moderate or high prevalence of HBV infection to clarify the precise impact of active immunization on the emergence of OBI in the vaccinated population. In addition, longitudinal studies on high-risk groups are beneficial to rule out cases with transient OBI. Finally, the development of new upgraded vaccines would be our trump card in the war against viral hepatitis.

## Funding

This research received no external funding.

## Consent for publication

Not applicable.

## CRediT authorship contribution statement

**Sara Delghandi:** Writing – review & editing, Writing – original draft, Investigation, Data curation. **Ramin Raoufinia:** Writing – original draft, Investigation. **Sahar Shahtahmasbi:** Writing – original draft, Investigation, Data curation. **Zahra Meshkat:** Writing – review & editing, Validation, Methodology, Investigation, Data curation, Conceptualization. **Hamed Gouklani:** Writing – review & editing, Validation, Conceptualization. **Aida Gholoobi:** Writing – review & editing, Validation, Supervision, Project administration, Methodology, Formal analysis, Data curation, Conceptualization.

## Declaration of competing interest

The authors declare that they have no known competing financial interests or personal relationships that could have appeared to influence the work reported in this paper.

## Abbreviations

OBI: Occult hepatitis B infection
HBV: Hepatitis B virus
